# Predictors of adherence to Hypertension Self-management practices among older persons attending a rural hospital in Uganda

**DOI:** 10.4314/ahs.v25i2.13

**Published:** 2025-06

**Authors:** Anna Kabona Mbaseege, Jane Frances Namatovu, Innocent Besigye, Sarah Kiguli

**Affiliations:** 1 Department of Family Medicine School of Medicine, Makerere University College of Health Sciences, Kampala, Uganda; 2 Department of Paediatrics and Chid health, School of Medicine, Makerere University College of Health Sciences, Kampala, Uganda

**Keywords:** Hypertension self-management, Uganda, chronic care model, sub Saharan Africa, Non communicable diseases

## Abstract

**Background:**

Hypertension self-management practices have proven beneficial in controlling blood pressure and preventing complications as part of the chronic care model. However, these practices among older persons 50 years and above have not been well evaluated in sub Saharan Africa despite the emerging hypertension burden in the region.

**Objective:**

The study determined adherence to hypertension self-management practices and factors associated with good adherence among older persons attending the Non-Communicable Diseases clinic in rural Eastern Uganda.

**Methods:**

In a facility-based cross-sectional study, individuals 50 years and above were selected consecutively from the bi-weekly hypertension out-patient clinic from January-February 2023. The adopted Hypertension Self-Care Activity Level Effects (H–SCALE) questionnaire was used to assess adherence to the self-management practices. The generalised linear model was used to determine predictors of adherence to the self-management practices.

**Results:**

Of the 74 hypertensive patients, level of adherence to the hypertension self-management practices of atleast 4 out of the 6 practices was 27 (36.5%). Adherence to diet, weight management, medication adherence, aerobic physical activity, non-smoking and recommended alcohol intake was 0(0%), 23(31.1%), 33(44.6%), 37(50.0%), 67 (90.5%) and 74(100%) respectively. Being married and having hypertension for less than 4 years were associated with good adherence p-value 0.01 and 0.03 respectively.

**Conclusions:**

Social factors like marriage and duration with hypertension were associated with better adherence to hypertension self-management practices. We recommend better implementation of the chronic care model to improve hypertension control in the community.

## Introduction

Over one billion people worldwide have hypertension with two thirds of those affected living in low to middle-income countries^1^. The hypertension self-management practices have been proven beneficial in reducing morbidity and mortality by controlling blood pressure^2^. These practices include medicine adherence, weight loss, 150 minutes of aerobic physical activity weekly, non-smoking, reduced alcohol intake and eating a Dietary Approaches to Stop Hypertension (DASH) diet^3^. Some prior studies found that the factors influencing adherence include age, sex, marital status, educational level, residence, knowledge of the practices, comorbidities and duration of disease^4^-^6^.

Several Sub-Saharan African studies have shown that majority of hypertensive patients had poor adherence to these practices with one Ethiopian study finding it at 29.9% and a Tanzanian study finding it as low as 19.7%^6^,^7^. Older persons make up 4.1% of the Ugandan population and age is a non-modifiable factor associated with hypertension^1^,^8^,^9^. Despite this, studies among this age group with hypertension are limited.

Tusubira et al, 2020 did a study in Nakaseke district, Central Uganda on self-care practices. Most respondents reported adherence to daily medication, recommended dietary changes, weight management, non-smoking and reduced alcohol intake^10^. But literature on the subject is limited in Eastern Uganda despite having the second highest prevalence of hypertension in the country at 26.4%^11^. This study therefore sought to determine the level of adherence to hypertension self-management practices and its predictors among older persons attending the Non Communicable Disease (NCD) clinic at Tororo General Hospital (TGH) in Eastern Uganda.

## Methods

### Study design, setting and population

A cross-sectional study was done in the NCD clinic at the outpatient department (OPD) of TGH found in Tororo district, Eastern Uganda. The clinic is run bi-weekly by two nurses supported by a doctor. It provides overall clinical care for hypertension and diabetes, laboratory services and patient counselling. The study included all older persons aged 50 - 100 years with hypertension for at least 6 months, started on antihypertensive medication and attending the NCD clinic during the study period. The critically ill patients who could not complete the questionnaire and older persons with cognitive impairment were excluded.

### Sample size

Kish Leslie formula (Kish leslie, 1965) was used to calculate the sample size. A 95% confidence interval was used taking a 5% level of precision. The prevalence of hypertension self-management practices in Tororo district was estimated at 50% since it was unknown.

Therefore, the calculated sample size was 385 participants. The clinic had 91 registered patients thus a correction formula for a finite population was applied and a sample size of 74 participants was obtained.

### Sampling procedure

Consecutive sampling was done on every hypertensive older person fitting the inclusion criteria. Participants were recruited every clinic day until the required sample size was obtained.

### Data collection

Data was collected from January - February 2023. Informed consent was obtained and an interviewer administered questionnaire was used. It was administered by two trained research assistants comprising of a nurse and an enrolled midwife after the consultation was done.

The questionnaire had two sections. The first section assessed social-demographic factors and patient characteristics. It had eleven items consisting of multiple-choice questions and open ended questions. The second section comprised of the Hypertension Self-Care Activity Level Effects (H-SCALE), a tool measuring adherence to the hypertension self-management practices. It was developed by Dr, Jan Warren-Findlow, who granted us permission to use the tool for this study^12^. It was developed and validated in North America and has been adapted for use in some African countries^4^,^12^-^16^.

The H-SCALE has six parts; medication adherence, physical activity, eating a DASH diet, weight management, reduced alcohol consumption and avoiding tobacco smoking. Medication had three items scoring 0-21 and adherence was 21. Diet had 11 items scoring 0-77 with 32 being low diet quality, 33-51 medium diet quality and adherence being 52-77. Physical activity had two items scoring 0-14 with adherence at 8-14. Non-Smoking had two items scoring 0-14 with adherence being zero. Weight management had 10 items with a score of 10-50 with adherence being 40-50. Alcohol intake was assessed using an existing measure, the 3-item, National Institute on Alcohol Abuse and Alcoholism (NIAAA) Quantity and Frequency Questionnaire. Adherence was a score of 14 for males and 7 for females in the previous seven days. Good adherence was at least four out of six subscales of the HSCALE with necessity of medication adherence^16^. The adherence level cut-offs used in this study for low/poor, moderate and high/good were <50%, 50-79% and ≥ 80% respectively. These cut-offs have been used by other studies^17^,^18^.

### Data analysis

The data collected was entered into Epidata and double checked to ensure no errors were made then later exported into Stata version 17.0 for analysis. Numerical data was summarized using means and standard deviations. Categorical variables were summarized as proportions. The generalised linear model regression analysis determined the factors associated with good adherence. Variables with a p-value of 0.2 or less and those with biological plausibility were the ones considered for multivariate analysis after bivariate analysis. A P-value of < 0.05 was used as a measure of statistical significance and results were presented using tables.

### Ethical considerations

Ethical clearance was obtained from the School of Medicine Research and Ethics Committee of Makerere University College of Health Sciences, reference number Mak-SOMREC 2022-371. Permission was also granted from the administration of TGH. Voluntary written informed consent was obtained from the study participants with privacy and confidentiality maintained. Covid 19 standard operating procedures were observed.

## Results

Almost a third of the study participants had good adherence to the hypertension self-management practices 27 (36.5%). All participants adhered to the recommended alcohol intake 74 (100%) and nearly all participants adhered to non-smoking 67 (90.5%). Half of the participants adhered to the recommended physical activity level 37 (50%) but less than half adhered to medicine prescription 33 (44.6%). Only a third of the participants adhered to the recommended weight management 23 (31.1%) and none could fulfil the diet recommendation. The results are further shown in table 1.

**Table 1:** Adherence levels to the hypertension self- management practices among older persons attending Tororo General Hospital, Uganda

Adherence levels (N=74)	Frequency (n)	Percentage (%)
**Overall adherence level**		
Good adherence	27	36.5
Poor adherence	47	63.5
**Hypertension self-management practices**		
Reduced alcohol intake	74	100.0
Avoiding tobacco smoking	67	90.5
Physical activity	37	50.0
Medicine adherence	33	44.6
Weight management	23	31.1
Diet adherence	0	0.0
**DASH diet adherence levels**		
low diet quality/low adherence	17	23.0
medium diet quality/moderate adherence	57	77.0
High diet quality/high adherence	0	0.0

The most significant factors on bivariate analysis were being married PR 95%, CI 8.98 (1.29-62.57) p=0.027 and not having hypertension for a longer duration CI 0.50(0.22-1.17) p=0.111.

On multivariate analysis, being married had a higher association with good adherence than being widowed/separated (p-value 0.010). Also having a hypertension diagnosis ≥4 years was less associated with good adherence levels (p-value 0.030) in comparison with those having hypertension for <4 years. These results are further shown in table 2.

**Table 2:** Analysis of the social-demographic and patient characteristics associated with good adherence to hypertension self-management practices among older persons attending Tororo General Hospital, Uganda

Characteristic	Poor (N=47) n (%)	Good (N=27) n (%)	PR (95%CI)	P-value	Adjusted PR (95%CI)	P- value
**Age (years)**						
50-60	28 (59.6)	12 (44.4)	1.00			
61-70	10 (21.3)	11 (40.7)	1.75 (0.93-3.28)	0.082		
≥71	9 (19.1)	4 (14.8)	1.03 (0.40-2.65)	0.958		
**Sex**						
Female	32 (68.1)	17 (63.0)	1.00			
Male	15 (31.9)	10 (37.0)	1.15 (0.62-2.14)	0.652		
**Marital Status**						
Widowed/separated	18 (38.3)	1 (3.7)	1.00		1.00	
Married	29 (61.7)	26 (96.3)	8.98 (1.29-62.57)	**0.027**	12.04 (1.76-82.37)	**0.010**
**Tribe**						
Japadhola/Iteso	38 (80.8)	25 (92.6)	1.00			
Others	9 (19.2)	2 (7.4)	0.46 (0.13-1.68)	0.239		
**Occupation**						
Peasant farmer	30 (63.8)	20 (74.1)	1.00		1.00	
Employed	12 (25.6)	4 (14.8)	0.63 (0.25-1.57)	0.317	0.46 (0.20-1.06)	0.068
No job	5 (10.6)	3 (11.1)	0.94 (0.36-2.46)	0.896	0.65 (0.25-1.68)	0.369
**Education level**						
No formal education/primary	35 (74.5)	19 (70.4)	1.00			
Secondary education/tertiary	12 (25.5)	8 (29.6)	1.14 (0.59-2.18)	0.700		
**Residence**						
Rural	29 (61.7)	13 (48.1)	1.00			
Urban/peri-urban	18 (38.3)	14 (51.9)	1.41 (0.77-2.58)	0.26		
**Duration with hypertension**						
<4 years	29 (61.7)	22 (81.5)	1.00		1.00	
≥ 4 years	18 (38.3)	5 (18.5)	0.50 (0.22-1.17)	**0.111**	0.42 (0.19-0.92)	**0.030**
**Comorbidities**						
Diabetes	37 (78.7)	25 (92.6)	1.00			
Others	4(8.5)	2(7.4)	1.31(0.46-3.72)	0.609		
None	6(12.8)	0(0.0)	1.41(0.33-4.15)	0.208		
**Education on self- management practices**						
Yes	41 (87.2)	26 (96.3)	1.00			
No	6 (12.8)	1 (3.7)	0.37 (0.06-2.35)	0.29		

CI, confidence interval; PR, Prevalence ratio

## Discussion

Adherence to the hypertension self-management practises among the study participants was generally low. Diet, weight management and medication usage were lowest. It was high for reduced alcohol intake, non-smoking and moderate for physical activity. The only factors the study found significantly associated with good level of adherence were marital status and duration with hypertension diagnosis.

The low level of adherence to hypertension self-management practices especially medicine usage, weight management and diet could be due to lack of adequate knowledge on how to effectively carry out the practises and a poor attitude^19^. It could also be due to barriers in availability and affordability of some of the medication^20^. Adherence to reduced alcohol intake and non-smoking is expected for this age group since people tend to reduce risky behaviours as they grow older^21^. The fact that Tororo is largely a rural African area, the physical activity was lower than expected in this study. The only factors associated with good adherence were marital status and duration of the hypertension diagnosis. Married people tend to have more social support from their spouses who encourage them to carry out the practises compared to the unmarried^22^. Having hypertension for a shorter duration can motivate one to carry out the practises but over time the motivation reduces which can explain the study findings.

A low level of adherence to the hypertension self-management practises is similar to other sub-Saharan studies^6^,^7^,^15^. In contrast, other Ethiopian studies found higher adherence^14^,^23^. The differences could be due to the age groups of the study populations and the bigger sample sizes used when compared to this study. Nearly all the participants were adherent to reduced alcohol intake and non-smoking which is in line with another Ugandan study that found high adherence to these two behaviours^10^. This could be due to cultural similarities in the study populations and similar geographical locations since majority of the participants lived in rural areas. Half the participants were adherent to the physical activities which differs from other studies done in Ethiopia that found lower levels^4^,^6^. This could be due to the different age groups used in the studies since the participant's ages of this study ranged from 50-90 years. Medication adherence was low which corresponds to another Ugandan study 24. But a study done in China found medicine usage among older persons at 60.1%^25^. These divergences could be explained by the differences in the social-economic status between the two countries thus making the medication more affordable, available and accessible. This study's findings show that adherence to weight management and diet was generally low which is similar to one Ethiopian study^4^. But another Ethiopian study found them higher^14^. This could be due to that study having a bigger sample size, different study populations.

Being married was significantly associated with good adherence which differs from other studies that did not find this factor significant^15^,^23^. Participants with hypertension for greater than 4 years were less likely to have good adherence compared to those with hypertension for less than 4 years. The findings in the study contradicts another study that showed that good adherence is higher in those having hypertension for longer than 4 years^26^.

These the other studies having bigger sample sizes and differences in the age groups of the study participants. Surprisingly, other studies found sex, age, occupation, education level, residence, having comorbidities and education to be significantly associated with good adherence, which is contrary to what this study findings^4^,^6^,^27^. This could be due to methodological differences in the studies like study populations.

The study strengths include being carried out at a primary care facility, an initial point of contact for hypertensive patients. The study was done among hypertensive older persons, a mostly neglected group in Africa.

The study limitations include using a tool that was not validated in the local setting but has been adapted for use in similar settings (Ethiopia), using a hospital-based study which increased chances of selection bias since the participants who present to the hospital are those already seeking care. Recall bias due to using a cross sectional study. We used a non-random sampling technique which might have affected the representativeness.

## Conclusion

Majority of the older persons in the study do not adhere to the hypertension self-management practices which are crucial in controlling blood pressure and prevention of related complications. Marital status and duration of disease were the only factors significantly associated with good adherence.

The study recommends implementation of the chronic care model. It is a multisystem approach which includes; leadership commitment to improving hypertension management and providing adequate resources for it. Patient empowerment to self-manage through health education and skills training on how to identify the reasons for non-adherence thus improving them and linkage to peer support groups. All these strategies will lead to improved sustainability in adherence since it reduces as the years go by. Future studies should use tools locally validated in the Ugandan setting with a larger sample size. A qualitative study is needed to further explain the findings.

## Data Availability

The datasets used and analysed during this study are available from the corresponding author on reasonable request.
